# Survival in a Case of Emphysematous Cholecystitis With Sepsis Caused by Clostridium perfringens

**DOI:** 10.7759/cureus.49705

**Published:** 2023-11-30

**Authors:** Yuki Hoshi, Kaoru Takeshima, Shinsei Matsuoka, Tatsuhiko Hoshikawa, Koji Senuma, Takeshi Nakamura, Masashi Tsugita, Makoto Nakamaru

**Affiliations:** 1 Surgery, Fussa Hospital, Fussa, JPN; 2 Surgery, Oyamadai Clinic, Ageo, JPN

**Keywords:** bacterial liver abscess, clostridium perfringens, gram-positive rods, bacterial sepsis, emphysematous cholecystitis

## Abstract

A 77-year-old man presented to the Department of Internal Medicine with a chief complaint of abdominal pain. During the outpatient examination, a computed tomography (CT) scan showed gallstones. The patient developed worsening abdominal pain and fever and was admitted to the emergency department. He was diagnosed with cholecystitis and hospitalized. Treatment with antimicrobial agents was initiated. On the second day of hospitalization, the patient developed a fever of 39°C, hypotension, and oliguria. An emergency CT scan was performed, which showed gas production in the gallbladder. He was diagnosed with emphysematous cholecystitis, and emergency percutaneous transhepatic gallbladder drainage was performed. The patient was transferred to the high-care unit, and intensive care was initiated. On the eighth day, a follow-up CT scan showed an abscess in the gallbladder bed, and drainage was performed percutaneously. His general condition gradually improved, and he was discharged from the hospital on day 24. The patient was readmitted for cholecystectomy three months after the initial admission. The prognosis of sepsis caused by *Clostridium perfringens* is extremely poor, with a mortality rate of 70%-100%. We present a case of emphysematous cholecystitis successfully treated with multimodal treatment despite the presence of sepsis due to *Clostridium perfringens* and discuss the possible prognostic factors by reviewing the literature.

## Introduction

Sepsis, a severe clinical condition with significant morbidity and mortality, is currently recognized as a substantial public health concern. The incidence of sepsis cases per year has been gradually increasing, potentially attributed to the aging population [1]. Especially, sepsis caused by* Clostridium perfringens* has a high mortality rate and an extremely poor prognosis. In this report, we present a case of emphysematous cholecystitis with sepsis due to *Clostridium perfringens*, which was successfully treated with multimodal treatment, and discuss the possible prognostic factors by reviewing the literature.

## Case presentation

A 77-year-old male patient was admitted with a chief complaint of abdominal pain. His past medical history included stroke, type 2 diabetes mellitus, and hypertension. His family history was unremarkable. Nine days before admission, he had visited the internal medicine department of the hospital with a chief complaint of abdominal pain and was diagnosed with cholelithiasis. At that time, a thorough examination was planned to be conducted on an outpatient basis. Nine days later, he was admitted to the emergency department due to worsening abdominal pain and fever and was hospitalized with a diagnosis of cholecystitis.

On physical examination upon admission, the patient was awake, alert, and oriented to person, time, place, and events. Body temperature was 39.7°C, heart rate was 109 beats per minute, blood pressure was 140/81 mmHg, and SpO_2_ was 94%-96% (room air). Furthermore, physical examination revealed a flat and soft abdomen, spontaneous pain over the abdomen, and no tenderness. Laboratory tests upon admission revealed elevated white blood cell counts and C-reactive protein levels. The hepatobiliary enzyme and bilirubin levels were also elevated (Table 1).

**Table 1:** Laboratory data on admission. WBC: white blood cell; RBC: red blood cell; Hb: hemoglobin; Ht: hematocrit; PLT: platelet; PT: prothrombin time; INR: international normalized ratio; FDP: fibrin degradation products; CRP: C-reactive protein; AST: aspartate transaminase; ALT: alanine transaminase; LDH: lactate dehydrogenase; ALP: alkaline phosphatase; γGTP: gamma-glutamyltransferase; CK: creatine kinase; AMY: amylase; T-Bil: total bilirubin; Alb: albumin; Na: sodium; K: potassium; Cl: chloride; Glu: glucose; BUN: blood urea nitrogen; Cre: creatinine; eGFRcre: creatinine-based estimated glomerular filtration rate

Hematology		Coagulation		Biochemistry			
WBC	11,700/µL	PT	13.5 seconds	CRP	17.17 mg/L	Na	133 mEq/L
RBC	329 × 10^4^/µL	PT activity	84%	AST	60 IU/L	K	4.69 mEq/L
Hb	11.5 g/dL	PT-INR	1.1	ALT	54 IU/L	Cl	98.8 mEq/L
Ht	32.10%	FDP	15.1	LDH	257 IU/L	Glu	322 mg/dL
PLT	208,000/µL			ALP	429 IU/L	BUN	30.3 mg/dL
				γGTP	104 IU/L	Cre	1.29 mg/dL
				CK	61 U/LCv	eGFRcre	42.2
				AMY	43 IU/L		
				T-Bil	4.67 mg/dL		
				Alb	2.8 g/L		

**Figure 1:**
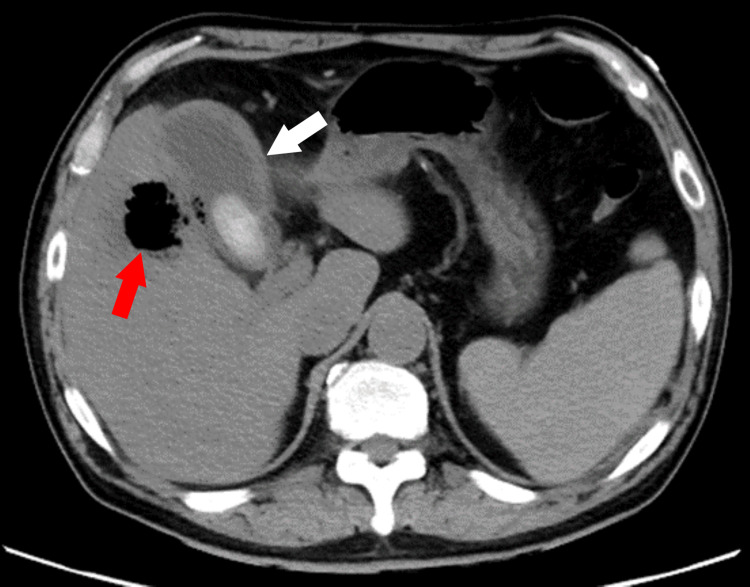
Abdominal CT scan demonstrating emphysematous cholecystitis (white arrow) and liver abscess (red arrow).

**Figure 2:**
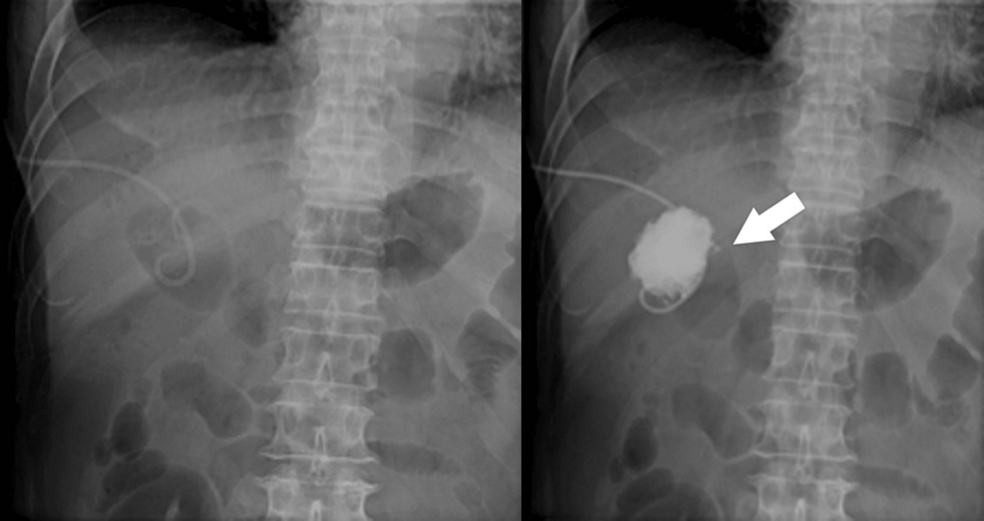
Percutaneous transhepatic gallbladder drainage. The cystic duct was not visualized, suggesting obstruction.

**Figure 3:**
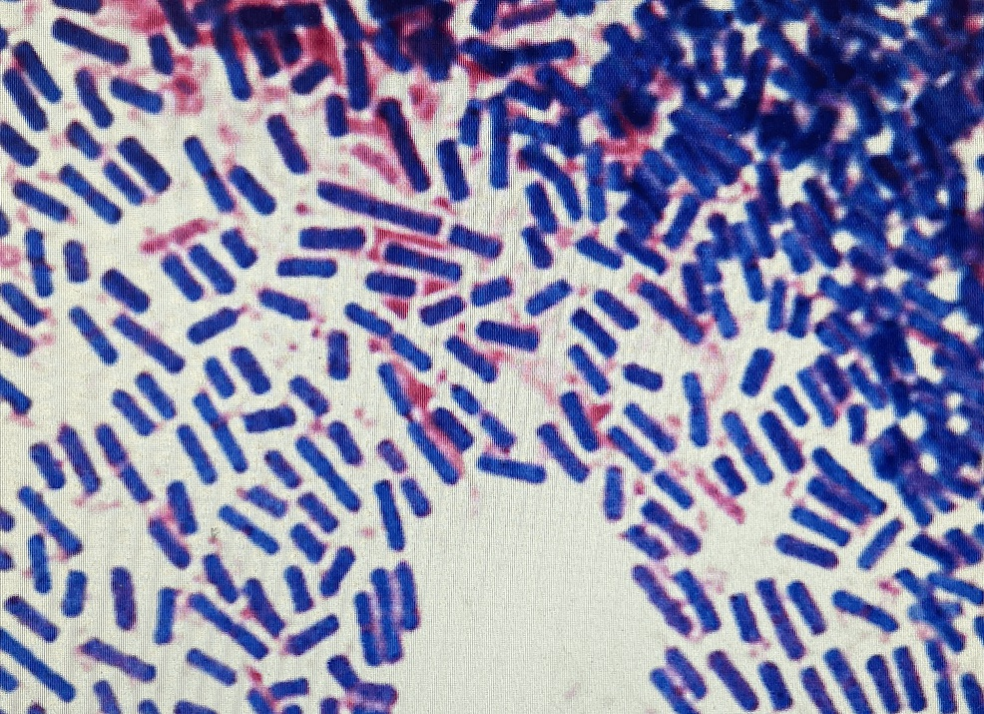
Specimen of blood culture (anaerobic bottle) revealing large Gram-positive rods.

On the second day of hospitalization, his blood pressure dropped to 90/58 mmHg, and he experienced chills and shivers. Blood tests showed a prominent elevation in white blood cells, C-reactive protein, hepatobiliary enzymes, bilirubin, and fibrin degradation products and a decrease in renal function (Table 2). A computed tomography (CT) scan on the same day revealed an enlarged, thickened wall gallbladder with a 4-cm gallstone impacted in its neck. Imaging also revealed a gas collection within the gallbladder wall and fluid and gas in the nearby liver tissue. Based on these findings, the patient was diagnosed with emphysematous cholecystitis and liver abscess (Figure 1). Percutaneous transhepatic gallbladder drainage (PTGBD) was performed after puncturing the liver abscess and aspirating the abscess as much as possible (Figure 2). At this time, Gram-positive rods were detected in the blood culture taken the previous day (Figure 3). The culture bottles were filled with gas, suggesting a large amount of gas production. Sepsis caused by *Clostridium perfringens* was suspected because of a quick Sequential Organ Failure Assessment score of 3 and a sharp increase of more than two points in the Sequential Organ Failure Assessment score.

**Table 2:** Changes in blood test findings between the first and second hospital days. WBC: white blood cell; CRP: C-reactive protein; AST: aspartate transaminase; ALT: alanine transaminase; LDH: lactate dehydrogenase; CK: creatine kinase; T-Bil: total bilirubin; BUN: blood urea nitrogen; Cre: creatinine; eGFRcre: creatinine-based estimated glomerular filtration rate

	First day	Second day
WBC (/µL)	11,700	42,900
CRP (mg/L)	17.17	19.06
AST (IU/L)	60	485
ALT (IU/L)	54	218
LDH (IU/L)	257	1,127
CK (U/LCv)	61	1,301
T-Bil (mg/dL )	4.67	19.42
BUN (mg/dL)	30.3	45.9
Cre (mg/dL)	1.29	1.87
eGFRcre	42.2	28.1

**Figure 4:**
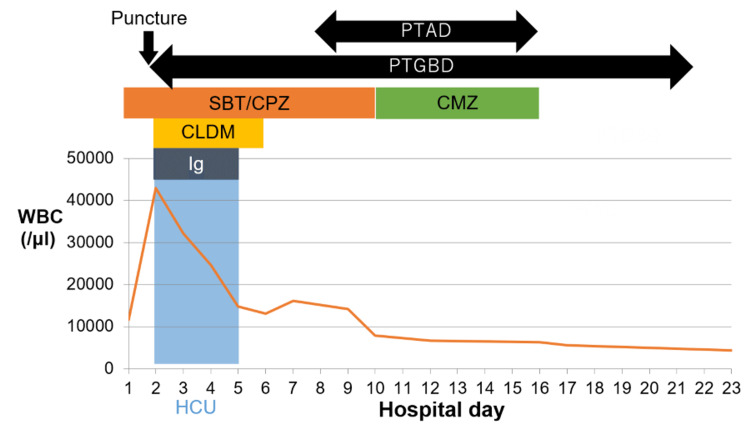
Progress of treatment. PTAD: percutaneous transhepatic abscess drainage; PTGBD: percutaneous transhepatic gallbladder drainage; SBT/CPZ: sulbactam sodium and cefoperazone sodium; CMZ: cefmetazole; CLDM: clindamycin; Ig: immunoglobulin; HCU: high-care unit

**Figure 5:**
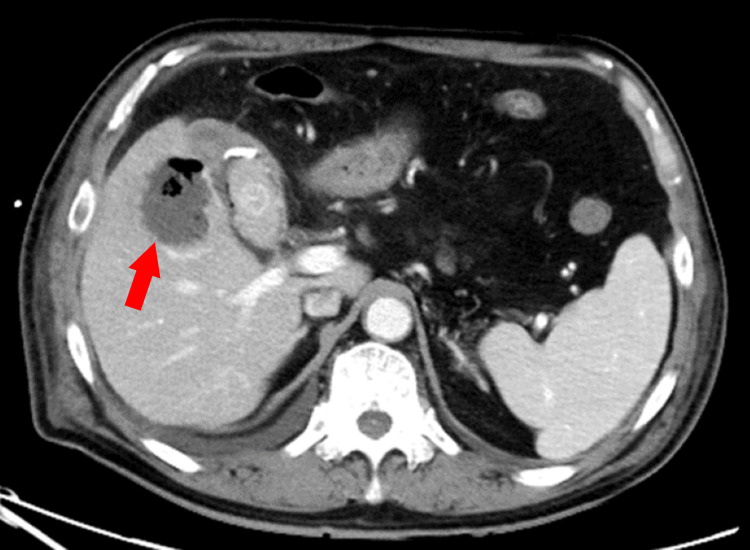
Abdominal CT scan showing fluid retention where there was originally a gas image of the liver abscess (red arrow).

**Figure 6:**
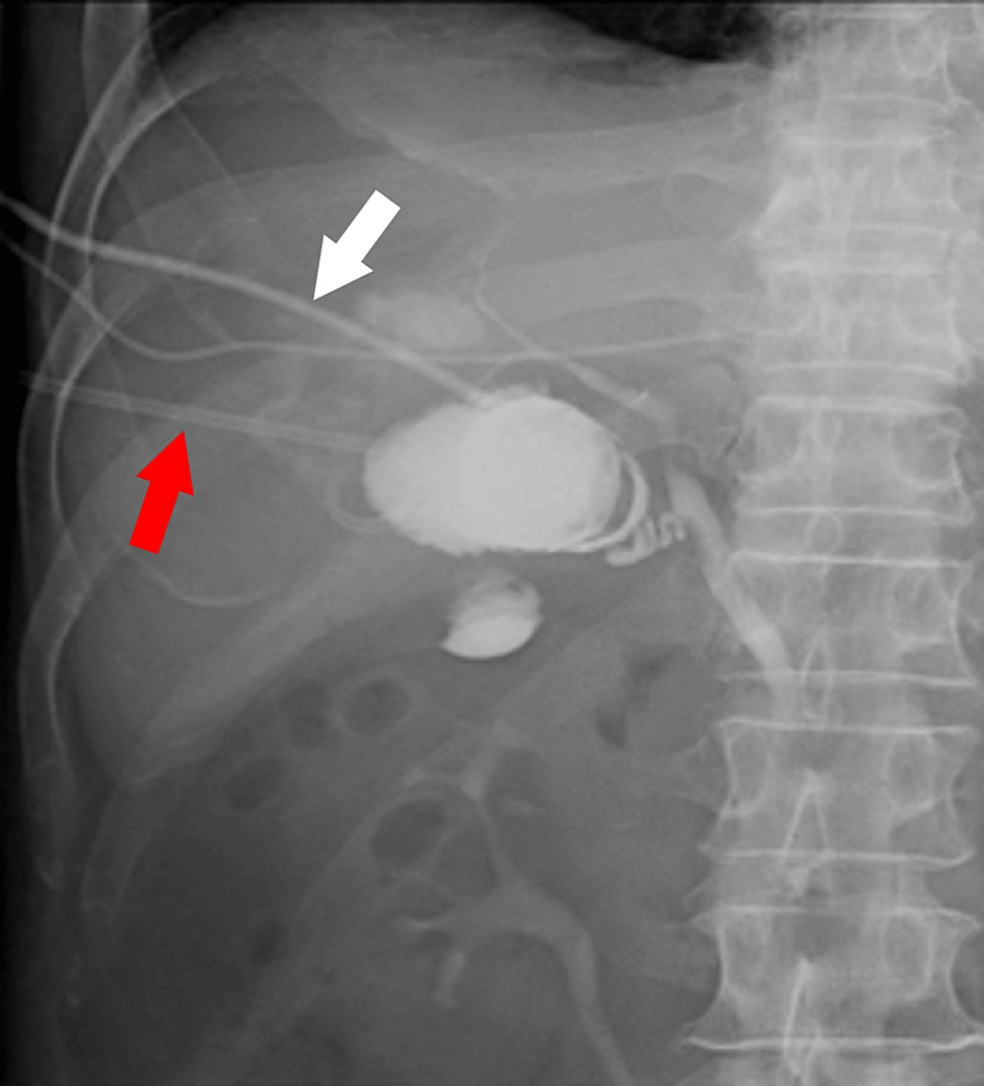
Percutaneous transhepatic abscess drainage (red arrow) was performed by puncture from the side of percutaneous transhepatic gallbladder drainage (white arrow).

The patient was transferred to the high-care unit on the second day and treated with cefoperazone/sulbactam and clindamycin. Additionally, he received gammaglobulin for three days for severe infection. Culture results were obtained on the sixth day, and *Clostridium perfringens* were detected in blood, pus, and bile. Based on the sensitivity test results, the antimicrobial agent was changed to cefmetazole on the 10th day (Figure 4). On the eighth day, a follow-up CT scan was performed, which showed fluid retention where there was originally a gas image of the liver abscess (Figure 5). Therefore, percutaneous transhepatic abscess drainage (PTAD) was performed on the same day by puncture from the side of PTGBD (Figure 6). Again, the abscess was sent for culture, but the culture was negative. On the 16th day, the PTAD was removed, and the PTGBD was removed on the 22nd day. The patient was discharged on the 27th day. Cholecystectomy was performed three months after admission, and subtotal cholecystectomy was performed due to severe inflammatory adhesion. Pathology revealed a diagnosis of xanthogranulomatous cholecystitis.

## Discussion

Emphysematous cholecystitis is a variant of acute cholecystitis caused by gas-producing organisms with gas images in the gallbladder and gallbladder wall. Diabetes mellitus, hypertension, and post-gastrectomy have been suggested as risk factors for emphysematous cholecystitis [2]. *Clostridium spp.*, including *Clostridium perfringens*, are the most common organisms causing emphysematous cholecystitis. Early treatment of emphysematous cholecystitis by taking *Clostridium perfringens* into consideration is important, even before the culture results are obtained [2].

*Clostridium perfringens* is an obligatory anaerobic Gram-positive rod bacterium. It is classified into types A-E according to the toxins produced. Type A is ubiquitous in the human gastrointestinal and urogenital tracts and mainly produces alpha-toxin [3]. This alpha-toxin is a lecithinase with phospholipase C activity that degrades phospholipids in cell membranes, causing hemolysis, tissue damage, and vascular endothelial damage.

Bacteremia and septicemia caused by *Clostridium perfringens* infections are most frequently due to hepatobiliary infections and mixed infections with *Escherichia coli* and *Klebsiella* [4]. The uterus and intestinal tract are other sources of infection. The fatality rate is 30%-40% in cases of bacteremia and 70%-100% in cases of sepsis, such as our case [5]. Treatment includes penicillin G and clindamycin, polymyxin B-direct hemoperfusion (endotoxin absorption therapy), and treatment of the underlying disease [6].

A literature search was performed in Ichushi and PubMed using the keywords “*Clostridium perfringens*” and “sepsis.” A total of 227 reported articles published from January 2000 to August 2023 were examined. Finally, 64 cases of hepatobiliary infection consisting of 63 reported cases and our case were compared based on survival (Table 3) [3,6-63]. The cases were divided into two groups based on survival, and factors, including age, sex, diabetes mellitus, cancer status, clindamycin use, and removal of infected foci, were compared (Table 4). Statistical analyses were performed using SPSS Statistics for Windows, version 29 (IBM Corp., Armonk, NY, USA). Statistical comparisons between the two groups were performed using the Wilcoxon rank-sum test or the chi-squared test. A p-value <0.05 was considered statistically significant.

**Table 3:** Reported cases of Clostridium perfringens sepsis due to hepatobiliary infection.

Author	Years	Age	Sex	Organ	Focus removed	Survival
Eckel et al. [7]	2000	65	F	Liver	Yes	Survival
Aoki et al. [8]	2000	83	M	Liver	No	Death
Otani et al. [9]	2004	73	F	Gallbladder	Yes	Death
Nakanishi et al. [10]	2004	68	M	Liver	No	Death
Kvitting et al. [11]	2005	77	F	Liver	Yes	Death
Kubota et al. [12]	2006	71	M	Gallbladder	Yes	Death
Loran et al. [13]	2006	69	F	Liver	No	Death
Ohtani et al. [14]	2006	78	M	Liver	No	Death
Eigneberger et al. [15]	2006	60	M	Liver	No	Death
Iida et al. [16]	2007	64	M	Gallbladder	Yes	Survival
Nukui et al. [17]	2008	72	F	Liver	No	Death
Meyns et al. [18]	2009	64	M	Liver	Yes	Death
Rajendran et al [3]	2010	58	M	Liver	Yes	Survival
van Bunderen et al. [19]	2010	74	M	Common bile duct	Yes	Survival
Kishi et al. [20]	2011	70	M	Liver	No	Death
Ch'Ng et al. [21]	2012	74	M	Liver	Yes	Death
Sato et al. [22]	2012	75	M	Liver	Yes	Survival
Kasimura et al. [23]	2012	74	F	Liver	No	Death
Qandeel et al. [24]	2012	59	M	Liver	Yes	Survival
Ingimarsidottir et al. [25]	2012	70	M	Liver	No	Death
Atia et al. [26]	2012	67	M	Common bile duct	Yes	Survival
Oshima et al. [27]	2013	74	M	Liver	Yes	Survival
Oshima et al. [27]	2013	70	M	Liver	Yes	Death
Bari et al. [28]	2013	48	F	Gallbladder	Yes	Survival
Cao et al. [29]	2013	59	F	Liver	No	Death
Kusumoto et al. [30]	2014	64	M	Liver	Yes	Survival
Kobayashi et al. [31]	2014	60	M	Liver	Yes	Death
Nakamoto et al. [32]	2014	75	M	Liver	No	Death
Wakasugi et al. [33]	2014	70	M	Liver	No	Death
Kitterer et al. [34]	2014	71	M	Liver	Yes	Death
Kurasawa et al. [35]	2014	65	M	Liver	No	Death
Haruki et al. [36]	2015	84	F	Liver	Yes	Death
Yoshida et al. [37]	2015	66	M	Liver	Yes	Death
Takada et al. [38]	2015	63	M	Liver	No	Death
Cochrane et al. [39]	2015	65	F	Gallbladder	Yes	Survival
van Dam et al. [40]	2016	86	M	Gallbladder	Yes	Death
Takahashi et al. [41]	2016	62	M	Liver	No	Death
Hashiba et al. [42]	2016	82	M	Gallbladder	No	Death
Lim et al. [43]	2016	58	M	Liver	No	Death
Carretero et al. [44]	2016	65	M	Liver	Yes	Survival
Kubo et al. [6]	2017	77	F	Liver	Yes	Survival
Kubo et al. [6]	2017	50	F	Liver	Yes	Survival
Kubo et al. [6]	2017	65	F	Liver	Yes	Survival
Tsukada et al. [45]	2018	65	M	Liver	Yes	Death
Harada et al. [46]	2018	54	F	Liver	No	Death
Geha et al. [47]	2018	74	M	Liver	No	Death
Saruwatari et al. [48]	2019	54	M	Liver	Yes	Death
Eto et al. [49]	2019	74	M	Liver	No	Death
Sakaue et al. [50]	2019	76	M	Liver	No	Death
Amjad et al. [51]	2019	77	M	Liver	No	Death
Chinen et al. [52]	2020	80	F	Liver	No	Death
Dahl et al. [53]	2020	68	M	Liver	Yes	Survival
Olds et al. [54]	2021	85	F	Liver	No	Death
Wang et al. [55]	2021	63	F	Liver	Yes	Survival
Woittiez et al. [56]	2022	65	M	Liver	Yes	Death
Takahashi et al. [57]	2022	70	M	Liver	Yes	Survival
Zhang et al. [58]	2022	74	M	Common bile duct	Yes	Survival
Lang et al. [59]	2022	60	M	Liver	Yes	Survival
Peng et al. [60]	2023	59	M	Liver	Yes	Death
Peng et al. [60]	2023	62	M	Gallbladder	Yes	Death
Tohmatsu et al. [61]	2023	72	F	Liver	Yes	Death
Osorio et al. [62]	2023	74	M	Liver	Yes	Death
Bayerl et al. [63]	2023	79	M	Liver	Yes	Survival
Our case		77	M	Gallbladder	Yes	Survival

**Table 4:** Comparison of the two groups.

	Survival group (n = 22)	Death group (n = 42)	P-value
Age (median)	65 (48‐79)	70.5 (54‐86)	0.16
Gender			0.63
Male	15	31	
Female	7	11	
Diabetes mellitus	12	20	0.6
Tumor-bearing state	6	11	0.93
Clindamycin	8	6	0.04*
Focus removed	22	18	<0.01*
Surgery	6	8	
Others	16	10	
*P < 0.05			

Of the 64 patients, 22 survived, and 42 died. The median age was 65 (48-79) years in the survival group and 70.5 (54-86) years in the death group (p = 0.16). In the survival group, 15 were males, and seven were females; in the death group, 31 were males, and 11 were females (p= 0.63). Diabetes mellitus was reported in 12 patients in the survival group and 20 in the death group (p = 0.60). Cancer was reported in six patients in the survival group and 11 in the death group (p = 0.93). Clindamycin was used in eight patients in the survival group and six in the death group (p = 0.04). All patients in the survival group and 18 patients in the death group had some form of removal of the infected lesions (p < 0.01). These findings strongly suggest that removal of the infection is a prognostic factor in the treatment of *Clostridium perfringens* sepsis. Regarding clindamycin, this result may be because clindamycin inhibits the production of exotoxins by *Clostridium perfringens* and other bacterial species [57]. The number of cases in both groups was small. Thus, more detailed studies with a more significant number of cases are needed.

This study has a limitation. Some of the death cases included in this study were in very poor condition, and the infected lesions could not be removed. However, all patients in the survival group had some form of removal of the infected lesions. We believe drainage or other forms of removal of infected lesions should be considered to save patients’ lives regardless of their general conditions.

## Conclusions

In this study, we reported a case of emphysematous cholecystitis with sepsis caused by *Clostridium perfringens*. In emphysematous cholecystitis, it is important to start the treatment with *Clostridium perfringens* in mind. Furthermore, we should actively consider removing infected lesions to save patients’ lives.
